# First report of the L1014F *kdr* mutation in wild populations of *Anopheles arabiensis* in Cabo Verde, West Africa

**DOI:** 10.1186/s13071-021-05088-4

**Published:** 2021-11-21

**Authors:** Derciliano Lopes da Cruz, Marcelo Henrique Santos Paiva, Duschinka Ribeiro Duarte Guedes, Elainne Christine de Souza Gomes, Silvia Gonçalves Pires, Lara Ferrero Gomez, Constância Flávia Junqueira Ayres

**Affiliations:** 1 Departamento de Entomologia, Instituto Aggeu Magalhães/Fundaçao Oswaldo Cruz (FIOCRUZ-PE), Av. Professor Moraes Rego s/n, Cidade Universitaria, Recife, PE 50670-420 Brazil; 2 Centro Academico do Agreste, Universidade Federal de Pernambuco, Rodovia BR-104, km 59—Nova Caruaru, Caruaru, PE 55002-970 Brazil; 3 Departamento de Parasitologia, Instituto Aggeu Magalhaes/Fundaçao Oswaldo Cruz (FIOCRUZ-PE), Av. Professor Moraes Rego s/n, Cidade Universitária, Recife, PE 50670-420 Brazil; 4 Universidade Jean Piaget (UniPiaget), Praia, Caixa 775 Cape Verde

**Keywords:** Malaria, *Anopheles arabiensis*, Knockdown resistance, *Kdr* mutation

## Abstract

**Background:**

Due to the lack of vaccines, malaria control mainly involves the control of anopheline vectors (*Anopheles* spp.) using chemical insecticides. However, the prolonged and indiscriminate use of these compounds has led to the emergence of resistance in *Anopheles* populations in Africa. Insecticide resistance surveillance programs are less frequent in Cabo Verde than in other African countries. This study aimed to investigate the circulation of the L1014F and L1014S alleles in natural populations of *Anopheles arabiensis* collected from two sampling sites in the city of Praia, Cabo Verde.

**Methods:**

*Anopheles* larvae were collected from the two sampling sites and reared in the laboratory until the adult stage. Mosquitoes were first morphologically identified by classical taxonomy and then by molecular species identification using molecular markers. All *Anopheles arabiensis* were subjected to PCR analysis to screen for mutations associated to resistance in the *Na*_*v*_ gene.

**Results:**

A total of 105 mosquitoes, all belonging to the *Anopheles gambiae* complex, were identified by classical taxonomy as well as by molecular taxonomy. Molecular identification showed that 100% of the *An. gambiae* senso lato specimens analyzed corresponded to *An. arabiensis.* Analysis of the *Na*_*v*_ gene revealed the presence of L1014S and L1014F alleles with frequencies of 0.10 and 0.19, respectively.

**Conclusions:**

Our data demonstrated, for the first time, the presence of the L1014F allele in the *An. arabiensis* population from Cabo Verde, as well as an increase in the frequency of the *kdr* L1014S allele reported in a previous study. The results of this study demonstrate the need to establish new approaches in vector control programs in Cabo Verde.

**Graphical Abstract:**

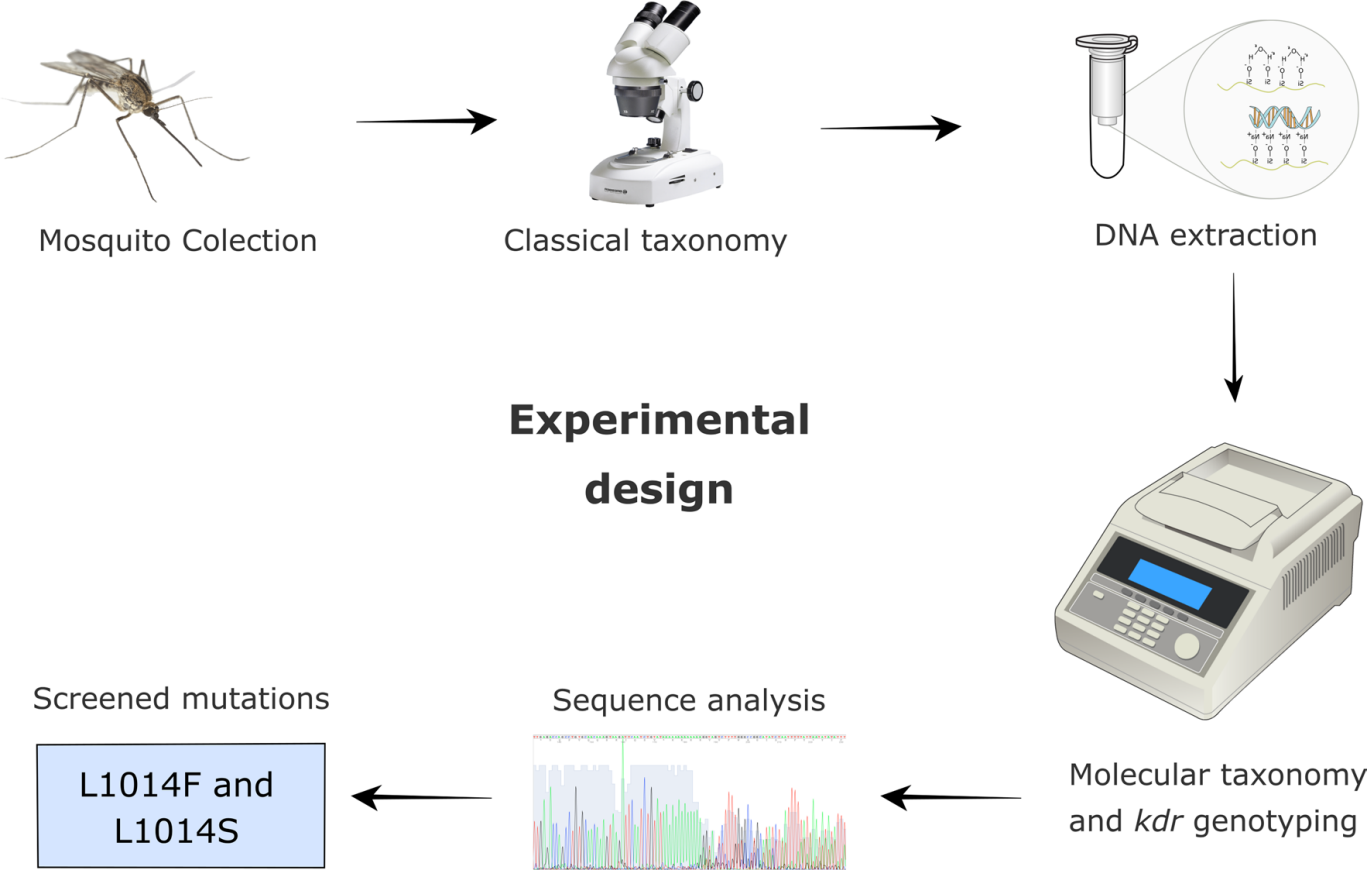

Malaria, caused by *Plasmodium* spp., is considered to be one of the most important vector-borne diseases from a global perspective. These parasites are transmitted by anopheline mosquitos (*Anopheles* spp.), and the highest world incidence is found in the African continent [1], where *Plasmodium* spp. are mainly transmitted by mosquitoes of the *Anopheles gambiae* complex [2–4]. Currently, this complex consists of at least seven cryptic species [5, 6], of which the most efficient are *Anopheles gambiae* sensu stricto (s.s.) and *An. arabiensis* [7–9]. In Cabo Verde, an archipelago located in the eastern Atlantic Ocean, off the coast of West Africa, the only species from this complex associated with disease transmission is *An. arabiensis* [10–14]. Malaria is considered to be endemic in the archipelago, although transmission has varied over the years. However, in the last 3 consecutive years there has been no local transmission in Cabo Verde; consequently, the archipelago is eligible to apply for the World Health Organization (WHO) certification of malaria elimination [15].

Malaria prevention relies mostly on vector control interventions based on the use of chemical approaches, such as long-lasting insecticidal nets (LLINs) and indoor residual spraying (IRS) [16, 17]. In Cabo Verde, since 1999, the vector control program has included the use of IRS with deltamethrin (an insecticide of the pyrethroid class) to control adult mosquitoes [18].

The extensive and indiscriminate use of chemical compounds has resulted in the emergence of a wide range of resistance in wild *Anopheles* populations, mainly in African countries [19]. Two mechanisms have been reported underlying resistance to xenobiotics in these populations: target-site insensitivity and metabolic resistance [20, 21]. Both mechanisms have already been documented in *An. arabiensis* in West Africa [22]. Target-site insensitivity in *Anopheles* is caused mainly by mutations present in the voltage-gated sodium channel (*Na*_*v*_) (domains II–IV) and they are commonly known as knockdown resistance (*kdr*) mutations. This particular mechanism has been reported in several studies conducted throughout the African continent [23]. *Kdr* mutations are among the best characterized point mutations and are often associated with resistance to pyrethroid (PYR) and organochlorine (OC) insecticides [24]. Two *kdr* mutations have been detected in the *Na*_*v*_ gene (at position 1014 of the encoded protein) of *Anopheles* populations: the replacement of leucine by phenylalanine (L1014F), found mainly in West Africa (the “*kdr-west*”), and the replacement of leucine by serine (L1014S), found mainly in East Africa (the “*kdr-east*”) [20, 25–27]. These mutations have been frequently detected in mosquitoes from African countries [28–33] and they have been used as a molecular marker for PYR resistance in *Anopheles* populations [34–36].

Information on the molecular mechanisms underlying resistance to xenobiotics in *Anopheles* populations is limited in Cabo Verde and, consequently, poorly understood in the archipelago. So far, a single molecular study has been carried out in Cabo Verde, which reported circulation of the L1014S mutation in local *An. arabiensis* [13].

The historical use of insecticides to control malaria in Cabo Verde reveals the necessity of finding molecular markers that may aid the detection and management of insecticide resistance in *An. arabiensis.* Thus, the present study aimed to investigate the circulation of the L1014F and L1014S alleles, often associated with resistance to pyrethroids, in natural *An. arabiensis* populations collected from Santiago Island, in Cabo Verde.

Cabo Verde is an archipelago of volcanic origin, composed of ten islands, located approximately 450 km off the West African coast, at the height of Senegal. The archipelago is characterized by its dry tropical climate and is inhabited by approximately 500,000 people [14, 37]. Approximately 60% of the entire population resides on the largest island of the archipelago, Santiago (991 km^2^), on which the capital city of Praia is located (Fig. 1) [38, 39]. This study was carried out in two neighborhoods of Praia: Achada Grande Trás (AGT; 23°29′12.22′′W, 14°55′11.85′′N) and Várzea (23°30′44.32′′W, 14°55′1.27′′N) (Fig. 1). The city of Praia is located on the southern coast of Santiago Island and is the economic and cultural center of Cabo Verde. Geographically, Praia may be described as a set of plateaus and respective surrounding valleys [40].

**Fig. 1:**
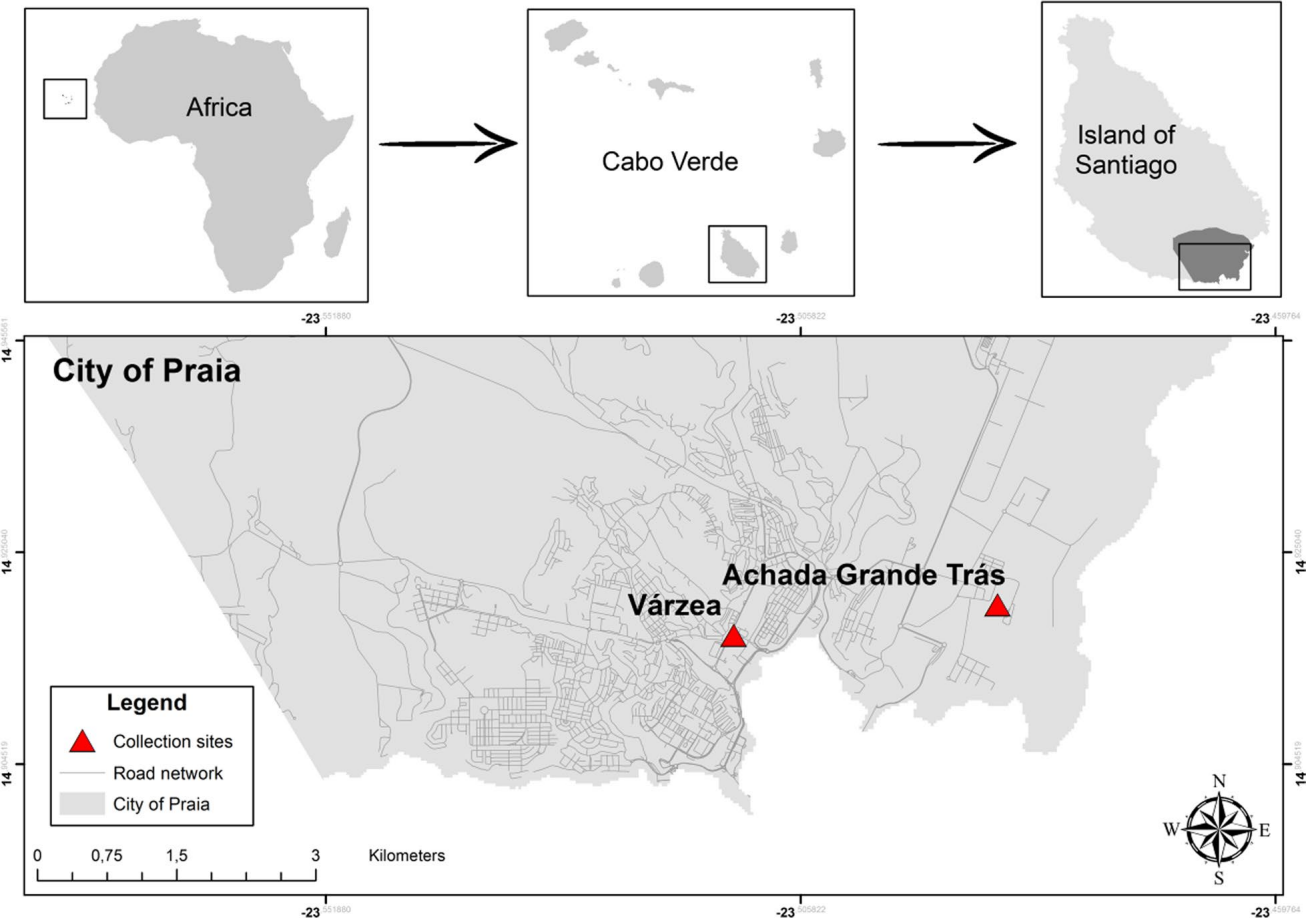
Geographic location of the Cabo Verde archipelago and collection sites. This map was edited in ArcGis, 2020

*Anopheles* larvae were collected in May and October 2017 (a single collection was carried out per month) in natural mosquito breeding sites, using an adapted 1-pint dipper. After each collection, larvae were sent to the insectary of the Entomology Department of Jean Piaget University of Cabo Verde and reared until the adult stage, under standard laboratory conditions (25–30 °C, 65–75% relative humidity and a 12:12-h light:dark cycle) [26, 41]. These specimens were then identified by classical taxonomy using the identification key of Ribeiro et al. [10]. Only mosquitoes from the *An. gambiae* complex were used for subsequent molecular analysis. Following morphological identification, these mosquitoes were preserved in 70% ethanol until DNA extraction and sent to the Entomology Department of the Aggeu Magalhães Institute (FIOCRUZ-PE) for molecular analysis.

Genomic DNA from individual mosquitoes was extracted following the protocol described by Ayres et al. [42] and the extracted DNA then stored at – 20 °C until analysis. Molecular identification of species was performed by PCR, according to the protocol described by Scott et al. [43]. DNA samples from *An. gambiae* sensu s.s., kindly provided by Dr. Maria Helena Silva Filha (Entomology Department at FIOCRUZ-PE), were included in each reaction as positive controls. After species identification, DNA samples were screened for the L1014F and L1014S *kdr* mutations, using a PCR protocol described by da Cruz et al. [13].

PCR products were sequenced using both forward/reverse primers at the Nucleus of Technology Platforms (NPT) of the FIOCRUZ-PE, using the Sanger method (Thermo Fisher Scientific ABI 3500xL genetic analyzer system; Applied Biosystems, Foster City, CA, USA). The CodonCode Aligner program (version 3.7.1) was used to check the quality of both sequences, as well as to edit and assemble the contigs (assembly criteria: sequences with  ≥  20 quality score were used to generate consensus sequences, based on the PHRED program). Sequence alignment and mutation identification were performed using the BioEdit program (version 7.2.6) [44].

A total of 105 *Anopheles* specimens belonging to the *An. gambiae* complex were identified by classical taxonomy and submitted for molecular species identification. The molecular analysis identified all individuals so analyzed as *An. arabiensis*, and these specimens were used to screen for *kdr* mutations. Of these 105 individuals submitted for *kdr-east*/*kdr-west* genotyping and sequencing, 91 resulted in informative sequences. Based on the sequences of these 91 mosquitoes, 10 were determined to be heterozygous (RS) and four homozygous (RR) for the *kdr-east* mutation (L1014S); 15 were heterozygous (RS) and 10 homozygous (RR) for the *kdr-west* mutation (L1014F); the remaining mosquitoes (*n * = 52) were homozygous for the susceptible genotype (SS) (Table 1).

**Table 1:** L1014S/L1014F genotype and allele frequency from *Anopheles arabiensis* collected in Praia City, Cabo Verde

Localities	*n*	Susceptible genotype	Allelic frequency	L1014S genotypes		Allelic frequency	L1014F genotypes		Allelic frequency
		SS	S	RR	RS	R	RR	RS	R
Várzea	26	17	0.69	1	1	0.06	6	1	0.25
AGT	65	35	0.71	3	9	0.12	4	14	0.17
Total	91	52	0.71	4	10	0.10	10	15	0.19

*AGT* Achada Grande Trás, *N* number of *Anopheles arabiensis* analyzed, *R* resistant allele, *S* susceptible wild-type allele, *RR* homozygous individuals for L1014F/S mutations, *RS* heterozygous individuals, *SS* susceptible homozygous individuals

The L1014S and L1014F allele frequencies were 0.10 and 0.19, respectively (Table 1). Although the two alleles were found in both collection areas, the L1014S allele was found more frequently in individual specimens collected in AGT, while the L1014F allele was found more frequently in those collected in Várzea (Fig. 2).

**Fig. 2:**
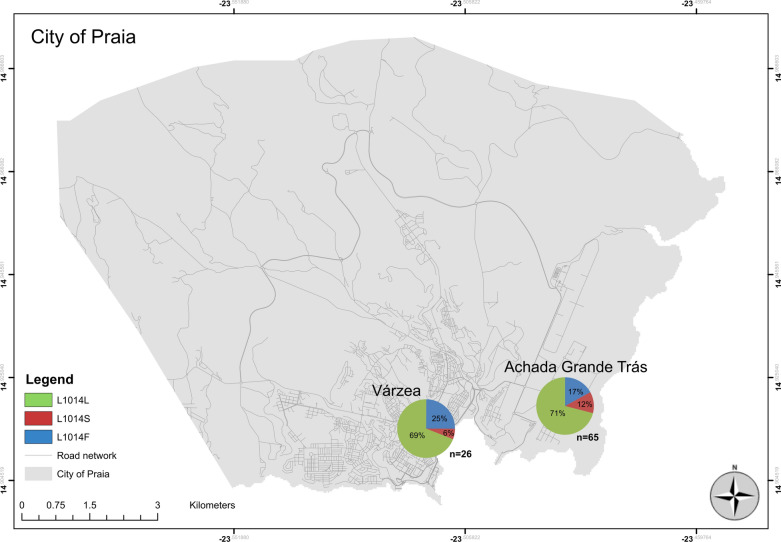
Allelic frequency distribution of the L1014S and L1014F mutations of the *Na*_*v*_ gene at the collection sites in Praia, Cabo Verde. *n* is the total number of individuals used to detect the L1014S and L1014F mutations in the *Na*_*v*_ gene

The surveillance of alleles associated with insecticide resistance in *An. arabiensis* populations is more frequent in other African countries than in Cabo Verde. However, Cabo Verde does not carry out surveillance in the same way as other African countries [45]. This study provides an update on the presence and frequency of *kdr* alleles in natural populations of *An. arabiensis* in Cabo Verde.

Our results demonstrate, for the first time, the presence of the 1014F allele in the *An. arabiensis* population from Cabo Verde. Although the sampling periods and localities differ from those in the study of da Cruz et al. [13], our data also show that the frequency of the 1014S allele (*kdr-east*) has increased, from 0.073 in da Cruz et al. [13] in samples collected in Cabo Verde in 2015, to 0.10 (present study). This difference shows that this allele is being gradually selected.

The *kdr-west* and *kdr-east* mutations have been found in several countries of the African continent, such as Burkina Faso, Côte d’Ivoire, Togo, Tanzania, Kenya, Senegal, Uganda, Ethiopia and Democratic Republic of Congo [24, 28, 30, 32, 33, 46–52]. This distribution indicates a dissemination of these two alleles across the continent as a consequence of gene flow between the different *Anopheles* populations and biogeographic regions [53].

In the present study, both mutations were found in the two study locations (AGT and Várzea), indicating that these mutations may be well distributed in Praia, where vector control based on chemical compounds is more frequent. However, a broader collection must be performed in other neighborhoods in the city of Praia to better estimate the allele frequency and their geographical distribution. As mentioned by da Cruz et al. [13], it is possible that the high frequency of alleles is associated with frequent use of pyrethroids in certain locations. More recently, temephos resistance was reported in *An. arabiensis* from the city of Praia [54], collected at the same time and at the same locations, which suggest a possible selective pressure for resistance to insecticides due to their greater use (frequency and quantity). Unfortunately, there is currently no public data on the use of pyrethroids by location to allow any estimation of a possible association between the use of chemical compounds on the island and the reported resistance data.

It is important to note that this is the second study related to mutations associated with molecular resistance in *An. arabiensis* carried out in Cabo Verde. These *kdr* mutations found in our study may be responsible for the resistance to deltamethrin 0.05% reported by DePina et al. [55] in the *An. arabiensis* population from the city of Praia. Those samples were collected during the same period that our samples were collected. The results suggest that the vector is adapting to the insecticides used in the local vector control program. The presence of these mutations represent a threat to vector control in Cabo Verde, since LLINs, a measure recently implemented by the Ministry of Health, contain deltamethrin, which could contribute to an increase in resistance to pyrethroids in the wild *Anopheles* population.

The results found in this study emphasize the need for frequent monitoring of the anopheline’s susceptibility to insecticides used in the vector control program in Cabo Verde. The WHO [23] recommends periodical monitoring of insecticide resistance in vector populations and that the monitoring process of insecticide resistance in malaria vector mosquitoes includes tests to determine the phenotype frequency (e.g. bioassays) and the mechanisms of resistance (e.g. molecular tests that determine the allele and genotype frequency). Unfortunately, in this study, it was not possible to perform bioassays to determine the phenotype of resistance to pyrethroids; therefore, we strongly recommend that in future studies, phenotypic analysis be performed followed by the determination of molecular mechanisms of resistance.

This study revealed an increase in the frequency of the *kdr* 1014S allele, when compared with previous findings, as well the presence of the *kdr* 1014F allele (identified for the first time) in *An. arabiensis* individuals collected on the island of Santiago. These results highlight the urgent need to create new vector surveillance strategies to establish new approaches in the vector control program in Cabo Verde. Molecular monitoring of resistance to chemical insecticides should continue to be carried out in order to guide the competent authorities in making decisions, such as the implementation of novel insecticides in the malaria vector control program in Cabo Verde.

## Data Availability

All data generated or analyzed during this study are included in this published article. The DNA sequences obtained here for each mutation were submitted to GenBank with the following access codes: RR (MW577186) and RS (MW577187) for the L1014F allele; and RR (MW577183) and RS (MW577184) for the L1014S allele. For the susceptible genotype (SS) the access code is MW577185.

## Abbreviations

AGT: Achada Grande Trás
IRS: Indoor residual spraying
*kdr*: Knockdown resistance
LLINs: Long-lasting insecticide nets
*Na*_*v*_: Voltage-gated sodium channel
RR: Homozygous individuals for L1014F/S mutations
RS: Heterozygous individuals
SS: Homozygous individuals
WHO: World Health Organization
